# An Evaluation of Graphical Formats for the Summary of Activities of Daily Living (ADLs)

**DOI:** 10.3390/healthcare8030194

**Published:** 2020-07-01

**Authors:** Caroline A. Byrne, Michael O’Grady, Rem Collier, Gregory M. P. O’Hare

**Affiliations:** 1 Department of Computing and Networking, Institute of Technology Carlow, Kilkenny Rd, Carlow, Ireland; 2 School of Computer Science, University College Dublin, Belfield, Dublin 4, Ireland; michael.j.ogrady@ucd.ie (M.O.); rem.collier@ucd.ie (R.C.); gregory.ohare@ucd.ie (G.M.P.O.)

**Keywords:** ambient assisted living, activities of daily living, design principles, usability, user interfaces

## Abstract

Activities of Daily Living systems (ADLs) and the User Interface (UI) design principles used to implement them empowers the elderly to continue living a normal daily routine. The daily monitoring of activities for most Assisted Living (AL) systems demands/necessitates accurate daily user interaction, and the design principles for these systems often focus on the UI usability for the elder, not the caregiver/family member. This paper reviews Ambient Assisted Living (AAL) and ADLs UI designs and evaluates the usability of ADLs visualisation tools for caregivers. Results indicate that the UI presenting information in a bar graph format was the preferred option for respondents, as 60% chose this summarisation method over the alternative line graph UI, which had 38% of respondents selecting this format for information representation. Therefore, when designing Ambient Assisted Living (AAL) UIs, it is recommended that short periods of time are best presented in a pie graph format in combination with a bar graph format for representing extended timeline information to caregivers about their loved ones.

## 1. Introduction

The changing demographic within the European Union presents a major future problem with those aged 65 years or over increasing from 17.1% to 30% by 2060 (84.6 million in 2008 to 151.5 million people in 2060) [1]. The United States in 1900 had a population of elders aged over 65 years at 4.1%, a century later it had tripled to 12.6% and is projected to reach 20% by 2030 [2]. In comparison, Europe and Japan [3] are predicted in the next twenty years to have unprecedented increases in elder population growth resulting from lower birth/death rates combined with better health care which has led to increased life expectancy. As documented by medical science [4], when humans reach an older age their health profiles often become more complex and their ability to sense accurately, disseminate information at speed and conduct various tasks simultaneously decreases dramatically. Furthermore, as we age, our ability to complete Activities of Daily Living (ADLs) in a timely fashion diminishes, and the burden of overseeing or verifying the completion of these daily tasks falls to caregivers.

The research documented in this paper is important as it investigates and highlights which User Interface (UI) is perceived by caregivers as easiest to understand when viewing summarised ADLs information in various graphical formats. Furthermore, it draws conclusions on which interfaces caregivers find the easiest to understand when viewing activity summaries depicted in various longitudinal timelines. These studies are important and serve to inform researchers when developing UIs for caregiver needs in order that the information being summarised is easily understood.

The research spotlight focused on Ambient Assisted Living (AAL) and ADLs systems has concentrated on User Interfaces (UIs) enabling elders to live independently, such as the MONAmI project encouraging social participation [5], the i2Home project UI [6], and kitchen ADLs UIs in the SWEET-HOME project utilising voice command for automating a home, along with The Smart Kitchen using speech activation; however, less attention has been attributed to the visualisation of ADLs for caregivers. The accurate monitoring and presentation of an elder’s daily activities in a graph format appropriate for different timelines which is easily understood by caregivers is difficult. This paper seeks to highlight how UI design and usability is critical for user uptake [7], in association with which graph format combination is the most easily understood by caregivers.

Nielsen’s 10 usability heuristics [8] and Shneiderman’s 8 golden rules [9] are well documented design principles, often applied in association with touch-based and multi-modal design principles [10]. Several research studies acknowledge that usability between younger/older adults is very different [11,12,13], that UI design for an older adult requires specific tailoring in order that the data can be processed efficiently; therefore, measuring usability for those over 65 years old should be quantified by virtue of a third (65–80 years) and fourth (80+ years) age group. This study reviews AAL and ADLs UI research designs and offers for comment a UI specifically for caregivers who seek unobtrusive reassurance that a loved one is completing their normal daily routine. Given the participant constituency for this research study was under 65 years, the issues for concern when designing UIs for the fourth age group (80+ years) did not concern this research.

When the terms user interface evaluation studies or the monitoring of ADLs in elders are researched, the results are predominantly populated with usability studies focused on providing the most intuitive UI for a system which enables elders to continue conducting their lives normally. Sensorisation studies which monitor elder’s ADLs, but do not offer for comment a caregiver UI for visualising generated data, include an unsupervised fuzzy approach to ADLs sensorisation [14] and the monitoring and early deviation detection with profuse sensor deployment [15]. Alongside smart home monitoring [16] are studies that monitor and recognize critical events, namely falls or extended inactivity by alerting caregivers with messages [17,18], or UIs displaying reports but do not offer for comment an interface visualising the collected elder information. This paper, in contrast, specifically reviews all ADLs UI systems, discusses the prominent visualisation methods adopted, and applies the most effective graph format combinations to ensure that caregivers understand the information being displayed. These interfaces applied the aforementioned graph formats in various combinations, and the usability of these UIs was evaluated by a cohort of caregivers through the use of two consecutive questionnaires conducted some nine months apart. This paper differentiates itself as it seeks to highlight the importance of caregiver UI design for the unobtrusive ADLs monitoring of elders and offers for comment several interfaces which incorporate previously researched graphical formats. Previous studies were conducted on smaller groups of respondents, whereas the participant cohort for this study was significantly larger than similar graph format evaluations, and this research concentrated on recruiting respondents who were caregivers, personally knew a caregiver or anticipated being a caregiver for an elderly relative in the future.

This paper is structured as follows: Section 2 discusses related works, such as a description of how humans interact with computers alongside a review of UI design within AAL and ADLs, specifically examining how daily activity information for the elderly is disseminated to caregivers. This review also investigates the visualisation methodologies previously proven in usability studies to be the most effective at conveying activity information. Section 3 outlines the methodology of how these activity summarisation formats are evaluated in this paper and which are the most effective at visualising different activity information timelines for caregivers. The study research questions and methodology are outlined, and a description of both questionnaires is provided. Section 4 discusses the statistical analysis methods employed and all results from both questionnaires. Section 5 indicates possible further research avenues, and finally the conclusion to this paper is discussed in Section 6.

## 2. Related Works

### 2.1. How Humans Interact With Computers

**Figure 1:**
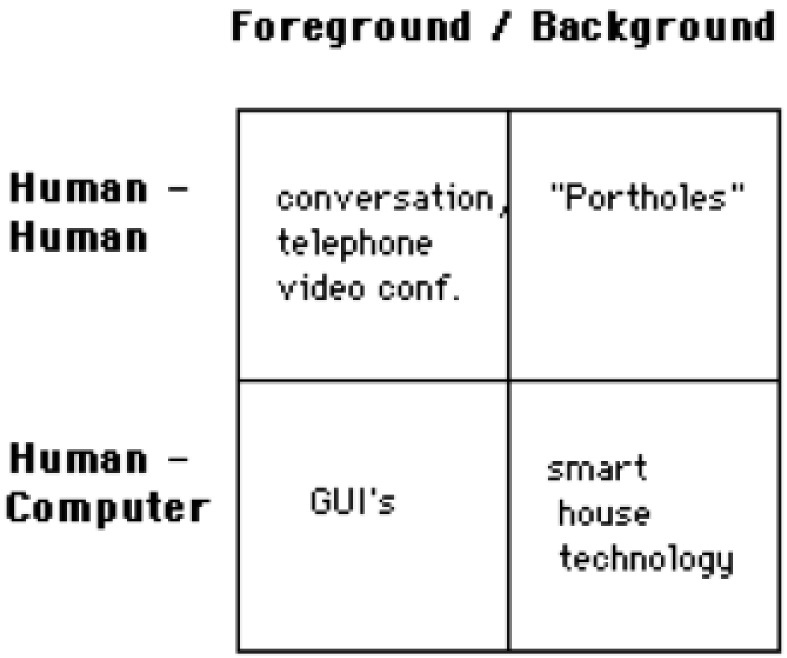
Buxton’s basic model.

Buxton’s basic framework in 1995 asserted that the subject areas of computing could be placed in categories [19], see Figure 1, incorporating a foreground and background, indicative of conscious and un-conscious tasks. AAL and ADLs systems rely on the technological agility of the user to manipulate interactions implicitly and explicitly, in the foreground when required [20] and otherwise in the background. These interactions ought to be ubiquitous, operating in the background as the user interacts in the foreground. UIs facilitating ease of use and effective Human–Computer Interaction (HCI) result in greater uptake [21,22].

**Figure 2:**
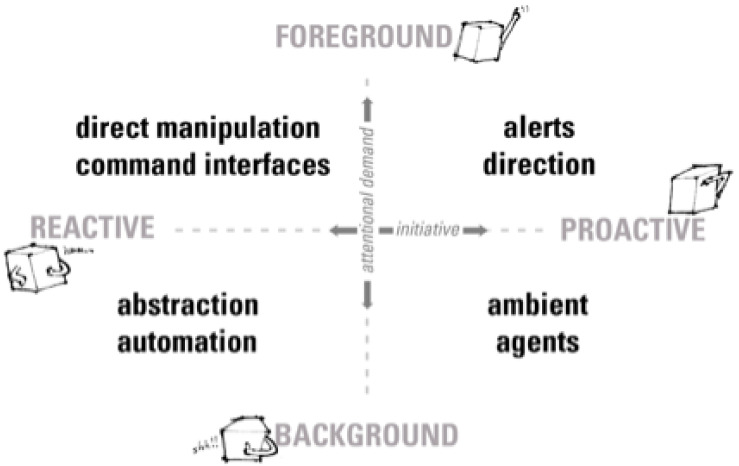
Implicit interaction framework [23].

Buxton’s matrix was expanded in 2008 based upon an axis of relevant criteria, see Figure 2, ref. [23] accommodating subtle, implied interactions that occur daily as humans and machines exchange information. Traditional computing can be implicit or explicit, and interactive exchanges are along a continuum that either demand a user’s attention with a command or it responds to a command initiated by the user [24]. AAL systems demand implicit and explicit communications, given users want unobtrusive systems, and achieving this balance of information exchange is the key to success. ADLs visualisation tools need to be in the attentional foreground if required by the user, otherwise to fade into the background. The theory behind Buxton’s original framework and the extended version contends that HCI can be plotted along a continuum of activities counter-balanced against the degree of invasiveness for the user. Critical to UI design emphasis in AAL/ADLs systems is the scales of balance for the user, an interface that assists when requested or when a scenario demands direct intervention is the goal. Systems that acknowledge UI design guidelines and incorporate frameworks have the ability to be truly effective in achieving that balance between user-initiated interactions and random un-requested system intrusions.

### 2.2. Assisted Living UI Review

Usability is fundamental to good UI design [25] as it facilitates the user executing their desired tasks, and this occurs if the interaction is intuitive and seamless. UI design must take cognisance of how humans interact with and process information whilst executing other tasks [26]. Ambient Intelligence (AmI) [27] has become intertwined over time with the computer science areas of engineering, biosciences and education, and the involvement of Human-Centered Computing (HCC) [28] has resulted in more effective methodologies being implemented. The most effective ADLs systems have utilised icons [29] for representing tasks such as ActivPal [30], ADLife [31] and Quietcare [32].

### 2.3. AAL and ADLs Systems UI Design Review

The AALIANCE AAL Roadmap advocates standardisation and adaptability in UI design frameworks [33,34] in order to enhance User eXperience (UX) for the elder user. Reviews of UniversAAL [35], research projects Soprano [36], Persona [37], Amigo [38], and Oasis [39] all highlight that UI/UX design in AAL/ADLs systems is a pivotal capstone. Further reviews of AAL/ADLs UI focus places the elder user centre stage with, for example, UI medicinal reminders. The Remote project [40], Tele-healthcare services [41], MonAMI [42], and Emerge [43] monitor and alert emergency services if there is a relevant ADLs deviation [44], yet no system offers an ADLs UI for carer/family member for their daily reassurance. The I-Living Project [45] developed an assisted living environment for embedded devices (sensors, actuators and displays), while the Home Sweet Home project in Ireland focused on elder UI usability for ADLs monitoring but not specifically for care-givers or relatives.

Caregivers of elders living alone constantly seek reassurance that normal daily tasks are being undertaken by their loved one. The unobtrusive supervision of ADLs with the assistance of various sensor types relaying real-time data to a UI has the capacity to offer reassurance. The methodology used to visualise a persons daily routines in a format that is easily understood is paramount to a successful uptake. Many displays comprise single-task representations, such as blood pressure levels [41], mobility or social interaction [40], while those that incorporate several features are often associated with AAL systems. This paper investigates how best to represent certain activities for caregivers in order to reassure them. The common ADLs often represented by such visualisation tools are those of sleep, medications, exercise, falls and localisation issues [36].

Sleep is a fundamental component in maintaining a healthy lifestyle. As we age, our medical needs become more complex, and sleep patterns may vary or become erratic. It is these variations that carers/relatives seek reassurance on [46] as it affects their ability to make decisions [47], reduces their mobility/dexterity and could lead to accidents within the home. The ability to take daily medications for extended periods of time is crucial for managing medical conditions, and non-compliance affects long-term health prognosis [48,49,50]. Mobility level is imperative for elders as it promotes agility, balance, health and well-being, reducing the risk of falling and subsequent health consequences. For caregivers, the ability to establish the whereabouts of a loved one through the use of localisation interfaces could be vital for reassurance and safety if dementia or Alzheimer’s is a limiting factor.

UI research indicates that numerical data, charts or icons are the popular choice for representing activities [51] within a calendar format. Buxton’s theory on foreground/background interactions are demonstrated as activities that comply with normal routine remain in the background, and deviations are brought to the foreground through colour differentiation. Another representation method is a dial or clock format [52] where either a circle or spoke represents data in regularised periods of time, again using colour to highlight areas of interest. Fall detection alerts [53] often via text message sent to the carer/relative provide an elder’s current location using a Uniform Resource Locator (URL) [54,55]. UIs not operating an aggressive alert system may result in falls remaining undetected for extended periods of time [56].

A review of care-giver UIs included HealthKiosk [57], an assistant for carer/relative of someone with dementia [58], GiraffPlus [59,60], home health monitoring to support carer/relative [61] and a home health feedback assistant [62,63]. The primary goal of these UIs is accurate daily task representation for carers/relatives, thereby enabling independent living and reassurance. Icons and charts such as pie, line or bar are used to display activity data in an easily understood format using colour to accentuate deviations from the norm. HealthKiosk uses the colour blue background with white buttons and task icons for ease of use and displays data in a line graph format. The UI for care-givers coping with someone diagnosed with dementia offers a red bar to highlight the emergency level and displays data in line graphs. GiraffPlus has a dominant yellow colour with a giraffe as its logo. Activities such as sleep are represented using a line graph with colours highlighting each daily task and the ability to view historical data for comparison. The home health system incorporates a mood icon or smiley face with the appropriate colour to represent the health status data of the elder in graphs (red, amber, green). Blue/grey are the dominant colours and also facilitate retrospective views of data for comparison purposes. The monitoring system UI is grey with tasks as different coloured buttons and deviations in historical data when viewed associated with a coloured background (red, amber, green) and graph format. Graphs, colour consistency and ease of use are common features of UIs depicting ADLs. The following subsection investigates which graph represents data in the easiest format for caregivers/relatives to comprehend.

### 2.4. Assisted Living UI Data Visualisation Methodologies

**Figure 3:**
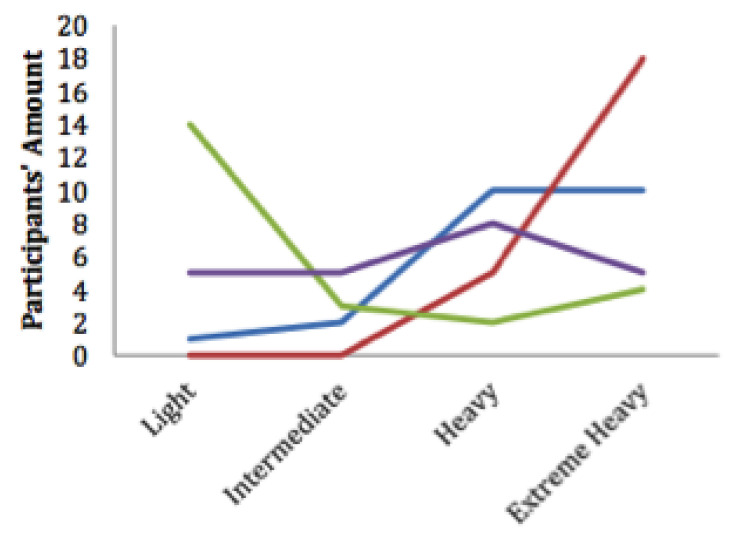
Line graph representation [53].

**Figure 4:**
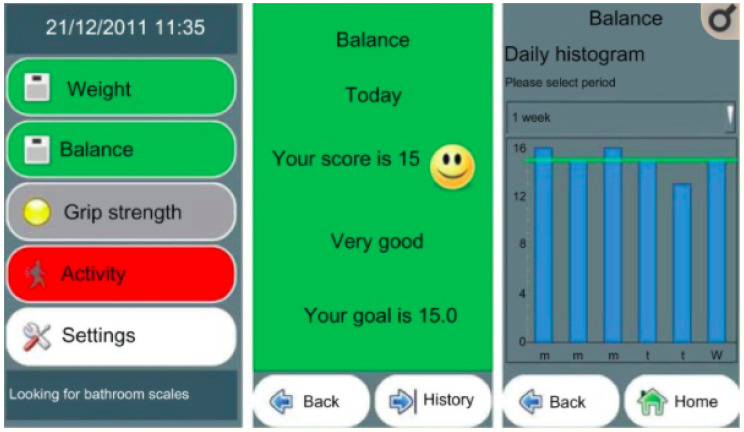
Bar graph representation [62].

**Figure 5:**
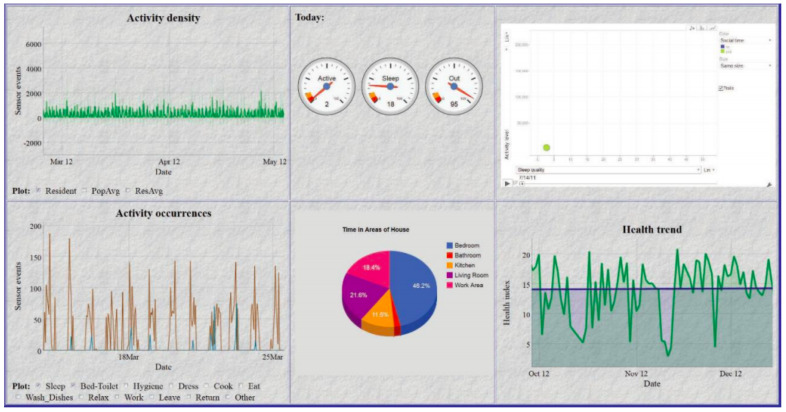
Pie graph representation [63].

**Figure 6:**
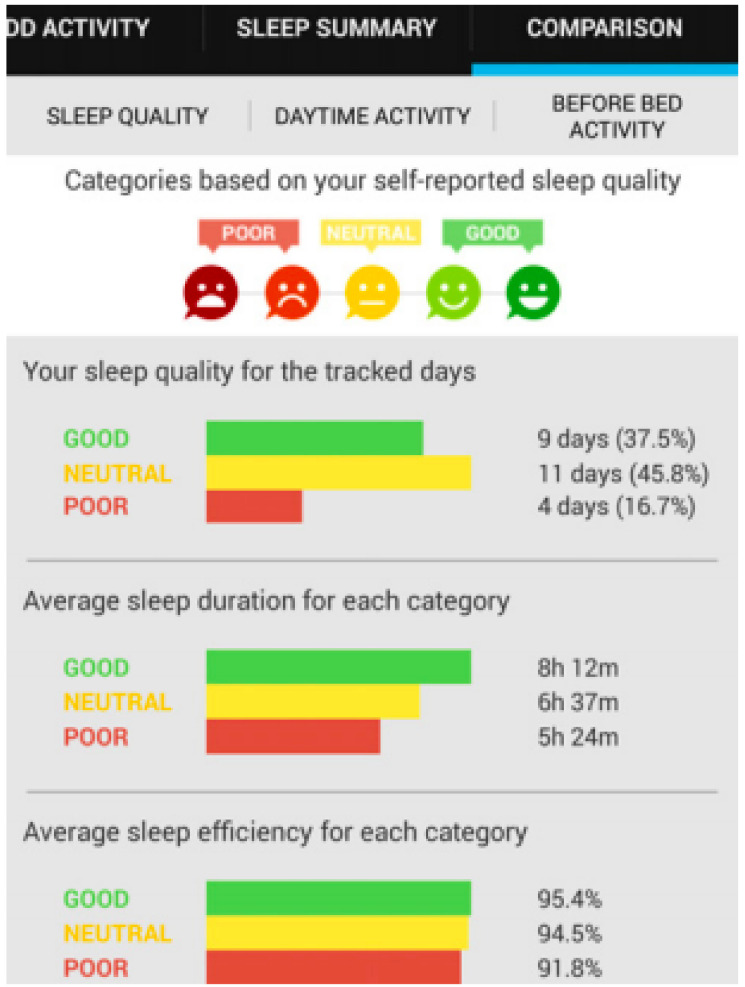
Combination format representation [66].

Research studies indicate that line, bar and pie charts, see Figure 3, Figure 4 and Figure 5, are the most easily understood [53,63,64] formats for displaying ADLs to users. A combination approach optimises the features from each format to maximise comprehension [65], see Figure 6. Line charts [66] can visualise the user requirements for easily understood UIs incorporating colour to accentuate areas of interest or concern.

The interfaces that implement a combination approach to data summarisation include UbiFit [64], which employs a glanceable display. This is a well-researched approach to visualising a day, or several hours of ADLs in a pie graph format as it is easier to assimilate, it is possible to glance at this format and understand the information it is summarising. SleepTight [67] depicts activities plus goal achievements in an easily understood visual format, and it presents sleep patterns via a glanceable widget display. Glanceable displays are intended for conveying information visually to the user with an icon or pie graph without demanding their full attention for comprehension of the data being visualised, see Figure 5. In contrast, whilst the visualisation of a longer time period is not easily understood with a glanceable display, it can be presented in a line or bar graph format for closer scrutiny as in Figure 3 and Figure 4.

Mobile phone technology is utilised for many ADLs studies [68,69], and the display often closely resembles one of the various data formats, see Figure 3, Figure 4 and Figure 5. The MobiSense tool [70,71] collected participants’ mobility statistics using mobile phone technology in an easy-to-use manner and displayed data in various formats depending on the time span chosen. A pie chart format visualises short intervals of time as a glanceable display, whereas longer periods of time when chosen are summarised as a timeline. Tong [72] experimented with three different formats for displaying user mobility data, bar chart representations from the FitBit dashboard, their circular ringmap and a virtual gamified pet. Results indicated the bar graph format was the most easily understood, and the ringmap represented time periods in a manner which assisted users in recognising their ADLs routines.

The visualisation of data in an easy to use and understandable format [73,74,75,76,77,78,79,80] is a complex task drawing on many research disciplines in order to work as a cohesive unit. Accurate ADLs recognition via a well-researched UI could prove an invaluable aid for the carers/relatives of an elder living alone, for the state as it grapples with the economic cost of an aging society and for our elders if it enables them to live longer within their own homes [81,82,83]. Many recent systems have focused on how appropriate visualision methods have influenced user uptake in mobility sensing [64] and to what extent mobile phones constitute a technologically robust method for data collection and subsequent uploading to a web service for report visualisation [68,69,70,71,73]. The focus of interface research has concentrated on visualisation tools for the elderly usually in association with a specific AAL system, and consequently current research within this area is inconclusive regarding which is the most effective mode for presenting easily understandable information to caregivers. This study, in contrast, pivots around caregivers and their need for unobtrusive, constant, timely reassurance that a loved one is conducting normal daily activities. It is imperitive that ADLs data are presented to caregivers in a format that is easily understood. This research seeks to resolve this issue for AAL designers everywhere by advancing UI design and establishing which graph format combination is the most effective for visualising information that is easily understood by caregivers.

## 3. Research Methodology for Caregiver UI Usability Studies

This paper reviews AAL and ADLs UI research design and investigates which data summarisation format is the easiest for caregivers to comprehend. This study sought to quantitatively [84] measure the usability of six interfaces with various graph format combinations which visualised a set of fictitious ADLs and were assessed using questionnaires [85,86]. The participant responses to both questionnaires were translated into usable statistics with the System Usability Scale (SUS) which quantified the attitudes, opinions and behaviour of the participants towards the UIs.

This research brings up front and central caregivers and their urgent need for unobtrusive monitoring of elders. This research addresses this caregiver need. It undertakes an appraisal of candidate UI usability and effectiveness in visualising ADLs data specific to caregivers. Activity information is displayed in various graphical formats for caregiver interpretation and evaluated through quantitative questionnaires. These usability studies contributed appreciably to discerning caregiver needs. It also exhibited novelty by recruiting the largest subject base to date for UI usability and efficacy of ADL information summarisation modes.

The interfaces in this study offer caregivers a ‘Virtual Window’ into an elder’s daily activities, providing reassurance and supporting longitudinal analysis. The long-term monitoring of ADL completion and the appropriate representation of such information supports caregivers in their role. AAL systems which embrace appropriate user-centric interface design principles are vitally important in facilitating the elderly in achieving their daily goals of health monitoring, social interaction, physical exercise or daily reminders, all of which are key to enhancing and prolonging independent living. This research explores the design of a personalised interface for the visualisation of a loved one’s daily activity completion. This usability study seeks to inform and shape the future design of a UI tailored specifically for caregivers. Previous studies conducted in this research space can be characterised by their small participation rates. This study, in comparison, comprised 66 and 64 participants, respectively, and is the largest study, to date, conducted on caregiver perceptions of interface usability.

The interfaces visualised a set of fictitious activities in formats demonstrated to be the most effective and easily understood by participants of research studies within this area. The ADLs chosen for representation [87] remained the same in all UIs, involving the household rooms in which the majority of daily activities are conducted, namely, the bedroom, bathroom, sitting room and kitchen. All interfaces had identical screen layouts, with consistent colour schemes [66], in order to focus the participant on the summarisation format being presented and not distract them with various layout preferences.

### 3.1. Aims and Objectives

- This research specifically sought to establish which UI was deemed by caregivers to be the easiest and most effective when remotely monitoring the daily activity information of loved ones.
- Current trends in user-centric applications for elder care support were investigated and used to inform the creation of a UI, not for the elder, but for caregivers who are often in a constant state of concern regarding the elder’s ability to continue living autonomously.
- This research on interfaces was specific to this category of user, that is, the informal caregivers, and it sought their opinion on which UI offered the most constant, informed and timely reassurance using easily recognisable icons to represent a summarisation of a loved one’s daily activity tasks.
- This study sought user insights in the form of various candidate interfaces summarising a set of fictitious daily activities. The UI displays were created in compliance with design principles [8,9,10,11] and with the User eXperience (UX) perspective foremost in design.

### 3.2. Sample

This research comprised of two usability studies, with the participants for both drawn from the same constituent base. This represented a continuity of caregiver opinion between both questionnaires. The participant sample base for questionnaire 1 was 66 and 64 for questionnaire 2. They comprised of members of the general community, complied with the inclusion criteria of being adults aged 18 years or over, having experience of caring for an elder and could give an informed consent. Both genders were represented equally within both studies. Whilst this study did strive for ethnic diversity, the opportunity to truly explore this avenue was limited given the studies were both conducted in rural Ireland where the constituent sample base is predominantly Caucasian. Participants had either some personal experience of caring for an elderly person, knew of someone caring for an elder, or may have to care for an elder in the future. The participants were recruited from the general community at public places, and written consent was gained after approaching them with information on the research study in the form of an information leaflet. Details of the study, what it involved, hoped to achieve, along with how to give and rescind written consent were included in the distributed information. Each study involved viewing specific interfaces and answering a usability questionnaire regarding that UI. The generated data were stored in UCD under REC Data Storage and Retention Guidelines in compliance with all General Data Protection Regulations (GDPR) guidelines.

### 3.3. Ethical Issues

There were no ethical dilemmas associated with this research as it did not seek the involvement of any vulnerable groups or request any participant involvement beyond a request to view UIs and answer a questionnaire designed to elicit demographic information regarding which graph format they felt was the most effective. Participants had experience of mobile phone technology, and occupations ranged from those in the health sector, Information and Communications Technology (ICT), clerical administration, professional carers for the elderly, business and social/childhood care.

Ethical approval was sought and granted from the office of Research Ethics, University College Dublin (UCD) for two separate UI usability studies. The aims and objectives for both usability studies were identical with the second study seeking further clarification on which of the final interfaces was the most usable, effective and easy to use.

**Q1: ADLs Usability Study** (University College Dublin Ethical Exemption Reference Number, LS-18-65, see Appendix A, for Questionnaire.)

1. This research sought to establish which UI is deemed by caregivers to be the easiest and most effective when remotely monitoring the daily activity information of their loved one.
2. To determine if an interface which combines two graph formats is deemed to be the most usable and effective by caregivers, the combination will include a pie graph visualising short-term daily activity analysis and a line or bar graph format for longitudinal activity analysis.

**Figure 7:**
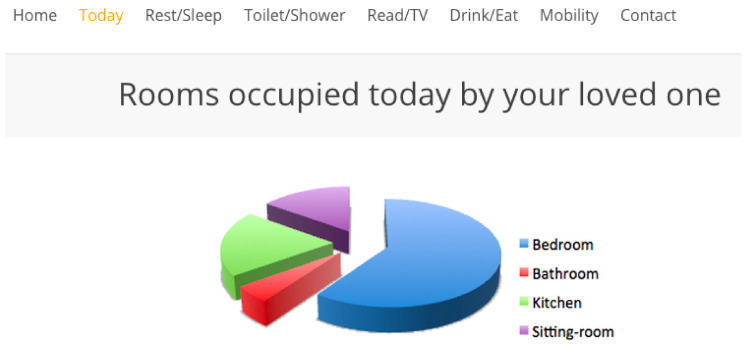
UI-1 pie graph format.

**Figure 8:**
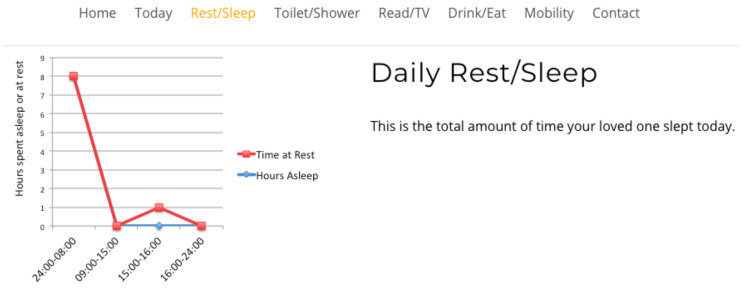
UI-2 line graph format.

**Figure 9:**
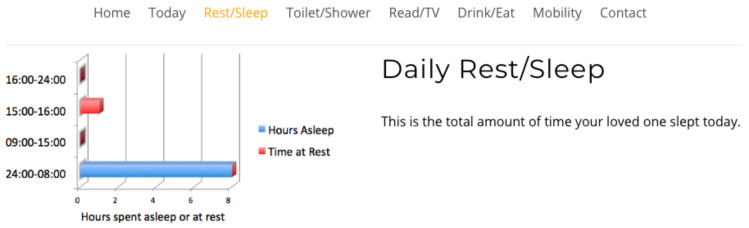
UI-3 bar graph format.

**Figure 10:**
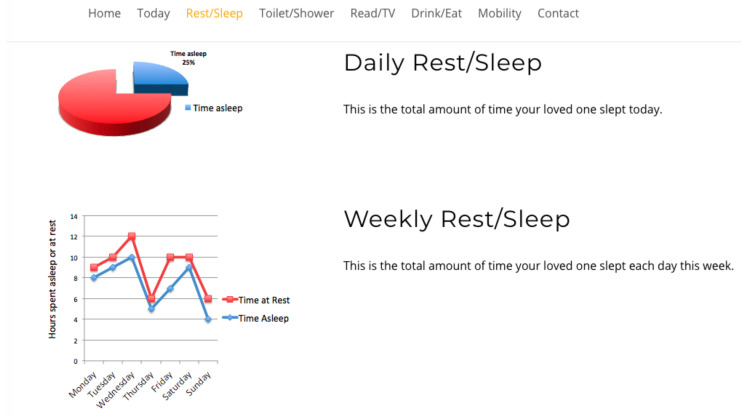
UI-4 combination graph format.

Study participants viewed four interfaces in succession, see Figure 7, Figure 8, Figure 9 and Figure 10, the first three exclusively summarised the data in only one graph format. UI-1 presented ADLs data in a pie graph format. Research indicates that a glanceable display for daily activities is the preferred option, see Figure 7 [64,65,72]. The second (UI-2), see Figure 8, and third (UI-3), see Figure 9, interfaces summarised data in a line then a bar graph format, which is commonly used for visualising extended time periods, and this aligns with previous research findings. The final interface, UI-4, featured all three graphical formats in combination to establish if this approach was the preferred method, see Figure 10 [68,69]. Participants were then asked to complete the SUS questionnaire [88,89] for each interface, which evaluated usability and ease of use. The final question in Q1 requested respondents to rank all four interfaces in order of preference.

When the data from questionnaire 1 was analysed, it was concluded that further scrutiny was required in order to achieve both study objectives. The first objective had been established as the UIs deemed by caregivers to be the easiest and most effective when remotely monitoring the daily activity information of their loved one were obtained. However, the results also indicated that the second objective had not been fully achieved; this sought to determine if an interface combining two graph formats was the most usable and effective for caregivers. A second review study was therefore required in order to gain greater clarity regarding exactly which graph format combination caregivers preferred for usability and ease of use.

**Figure 11:**
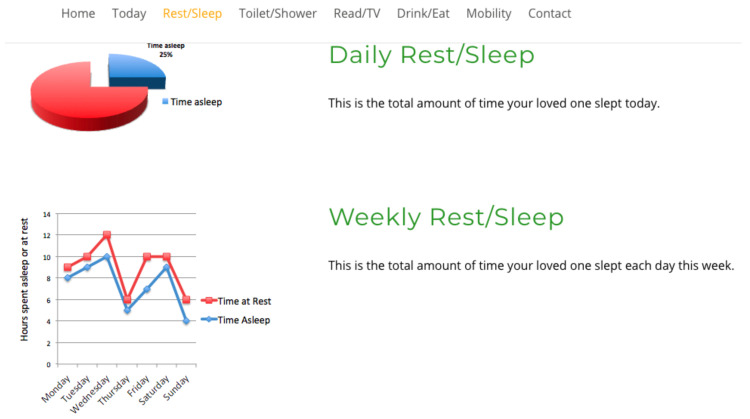
UI-5 pie/line format.

**Figure 12:**
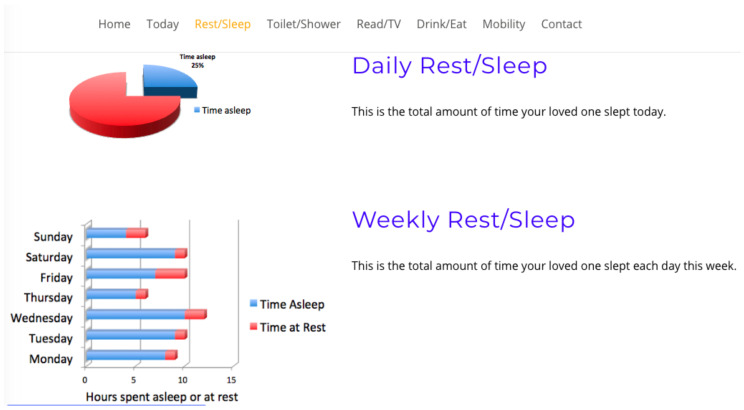
UI-6 pie/bar format.

Therefore, a second, subsequent questionnaire was conducted in order to achieve clarity on which of the two graph combination formats, pie/line or pie/bar which were presented to caregivers was deemed the most effective. The second study clarified interface usability, gathered demographic information on participant age profile, gender, experience of caring and confirmed whether the ADLs information being presented was understood by caregivers. The graph combination format presented to caregivers in questionnaire 2 was determined by the caregiver UI ranking choices from questionnaire 1. Questionnaire 2 repeated the same activity data as questionnaire 1 but within two graph combinations: UI-5 presents ADLs data in a pie/line graph format, see Figure 11, and UI-6 visualises data in a pie/bar graph format, see Figure 12.

**Q2: ADLs Usability Study** (University College Dublin Ethical Exemption Reference Number, LS-19-67, see Appendix B, for Questionnaire.)

1. This question gathered demographic information on participants, such as, age range, gender, IT and caring experience of elders for statistical purposes.
2. It also sought to determine if a combination graph format is deemed the most effective by caregivers, and which combination was selected by the majority of caregivers as their preferred choice of interface. Which of the two combinations offered for comment were the preferred choice, pie and line graph or pie and bar for summarising and visualising daily and longitudinal activities.

### 3.4. Methodology and Statistical Analysis (t-Test)

Data analyses were undertaken using SPSS v 24, and multivariate analyses, t-tests, and chi-square tests, including Fisher’s exact test, were all harnessed for interpreting feedback. An initial population of 69 for Q1 and 71 subjects for Q2 completed the questionnaire. During a preliminary screening of the dataset, three in Q1 and five subjects in Q2 omitted to complete the questionnaire satisfactorily. These subjects were removed, resulting in a final valid population of 66 (n = 66) for Q1 and 64 (n = 64) for Q2. Six questions were identified as remaining unanswered in Q2, and these were marked as such in SPSS.

#### Scoring Methodology for System Usability Scale

**Figure 13:**
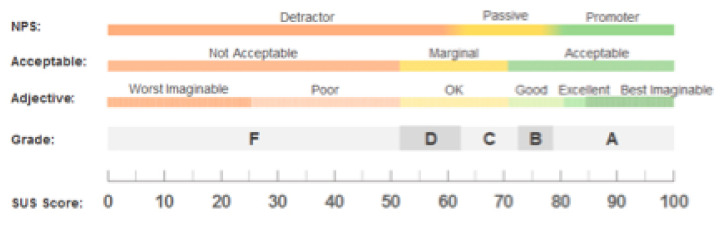
SUS Scoring Methodology.

The participants answered the SUS questionnaire on each UI and indicated from 1–5 their level of agreement or disagreement with the sentiment expressed in the question being evaluated. The second review questionnaire had an additional 11 questions included in order to gather demographic information on participants and if they actually understood the information being presented to them in the UIs. The SUS usability questions had a scoring methodology as follows: each odd-numbered question had 1 subtracted from the score and each of the even-numbered questions had their value subtracted from 5. At the end of each questionnaire, the total score was added and multiplied by 2.5. This resulted in a score out of 100, which is not a percentage but a percentile evaluation of system usability. The average SUS score was 68; if the UI score was under 68 there were issues with usability, if above 68 then this was within acceptable limits of usability, see Figure 13.

- A = 80.3 or higher means a high UI usability score;
- C = 68 or approximately means improvements could be made with the UI;
- F = 51 or under renders usability a serious issue with the UI.

## 4. Results

This section discusses the results derived from the statistical analysis of both study questionnaires. The data gleaned from both usability studies are discussed, their participant involvement numbers are outlined, and a statistical analysis of both questionnaires is examined in detail.

### 4.1. Questionnaire 1

**Figure 14:**
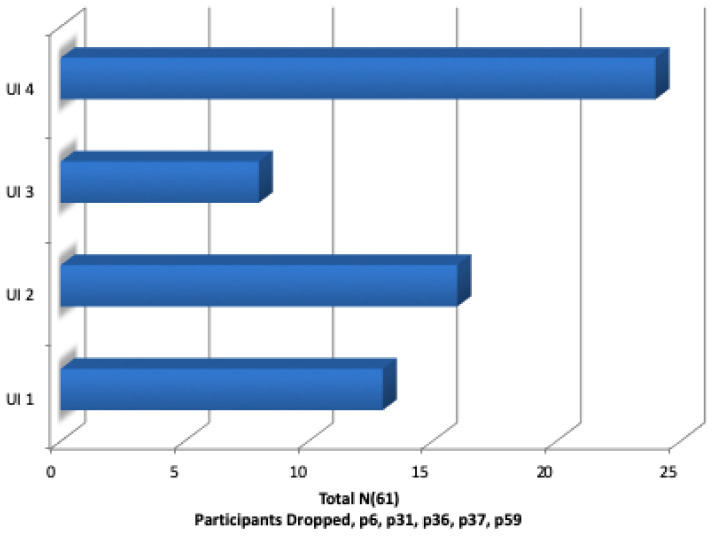
Q1 respondents rank 4 UIs.

This research study requested the 66 participants to initially view four UIs, then answer a questionnaire in order to elicit the respondents perception and usability of each interface. The final question requested that all four interfaces be ranked in order of ease-of-use and usability, see Figure 14. The quantitative SUS questionnaire was chosen for its simple format, long established academic reputation, and its 10 questions all address different aspects of the participants reaction to the interface as a whole. This was invaluable in measuring the reaction of participants to UI-4, which was a combination of a pie graph for daily activities and an extended timeline display for longitudinal ADLs analysis, namely a line or bar graph. The objective of this research was to establish which graph combination was the preferred display option for ADLs summarisation as chosen by caregivers. Research studies indicate that line, bar and pie charts, see Figure 3, Figure 4 and Figure 5, are the most easily understood [64,65,72] formats for long and short periods of time.

**Figure 15:**
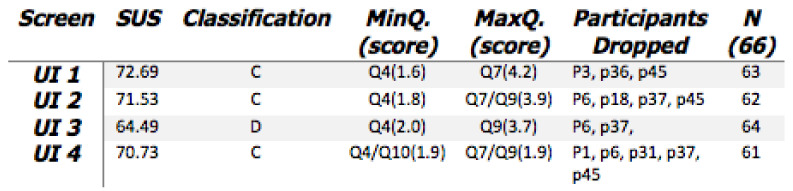
Q1 SUS results.

When the results from this questionnaire were analysed, it was decided that a definitive conclusion could not be reached on the issue of which interface was the preferred 1st choice by the majority of participants, see SUS scores in Figure 15. Therefore, a second review questionnaire was required seeking clarity regarding which interface was the most effective as perceived by caregivers. The following is a detailed analysis of the SUS participant responses to interfaces 1–4, followed by a final question requesting participants to rank all four in order of ease-of-use and preference. From the results, see Figure 15, the following inferences can be made.

The conclusion was reached that an area for concern still remained with regard to which interface was the most usable.

**Q1: Analysis of SUS Results**

- What is established from the results are some agreement on Q4 and Q7.
*Question 4: I think that I would need the support of a technical person to be able to use this website? Question 7: I would imagine that most people would learn to use this website very quickly?*
- When participants were requested to rank their UIs in order of preference for ’Ease of Use’, UI-4 was chosen by the most participants as their first preference.
- However, the SUS scoring system did not affirm this, with UI 4 only receiving an overall score of 70.73.

The results confirmed previous research findings that caregivers prefer a combination approach when viewing data, and this aligned with other research findings in this area, that caregivers prefer a combination approach for viewing different timeline data. Previous research studies confirm that bar or line graphs are the easiest to understand when viewing longitudinal time periods such as a week, month, year, and pie graphs and glanceable displays are the easiest to understand when viewing data presenting a short period of time, such as one day or part of a day. However, the results could not establish with a degree of certainty which graph combination the respondents preferred and was the easiest to understand. Hence, a review questionnaire was conducted to establish which of the combined graph formats was the preferred option, line or bar graphs, for long periods of time in combination with pie for short periods of time.

### 4.2. Questionnaire 2

The review research study requested 64 participants to view a further two UIs and subsequently answer a questionnaire in order to elicit usability. The second questionnaire offered for comment two UIs visualising daily activities, for which the short timeline data were displayed in a pie graph format, and the longer timeline data were represented in either a pie or line graph format, see Figure 11 for UI-5, with UI-6 presenting data in a pie/bar graph format, see Figure 12. Each UI displayed the same fictitious data as the first questionnaire, and the background display and UI layout remained identical. The only differences between these interfaces was the colour assigned to each, and the combination approach, pie/line (green) or pie/bar (blue). The colour coding of the interfaces was applied in order to remove any bias from a participant’s preferred choice of graph format combination. Analysis from the first questionnaire indicated that the majority of participants chose the bar graph format as their preferred UI; however, this was not affirmed by the SUS score. Therefore, the objective of the review questionnaire was to establish which graph format combination is the preferred choice of the majority.

Fundamental to the choice of statistical method is the notion of normal distribution. In summary, a normal distribution enables the adoption of parametric approaches, whilst a distribution that is not normal demands the use of non-parametric methods. Each approach has its merits and pre-conditions. However, a key advantage of parametric approaches is that they have more statistical power than the non-parametric approaches. Thus, they are more capable of leading to the rejection of the null hypothesis. Hence, it is often adopted for data that do not conform to a normal distribution [90]. In the case of the t-test, the population must be relatively large (n > 50) due to the Central Limit theorem; furthermore, skewness should be within the (–1, +1) and kurtosis between (–2, +2). For categorical data, the non-parametric chi-square is a preferred approach; however, this test depends on approximation. For smaller sample sizes, and particularly when more than 20% of cells have expected frequencies of less than 5 (<5), Fisher’s exact test is harnessed to test the association between variables. Where there are two or more dependent variables, a multivariate analysis is undertaken.

#### 4.2.1. Reliability

In the first instance, an assessment of the reliability of the questionnaire was undertaken using the standard SPSS Reliability Analyses feature. Most items were deemed worthy of retention; however three items were removed to improve reliability, resulting in a Cronbach alpha of 0.73.

#### 4.2.2. Demography

The gender profile of the group was almost equally divided, 51.5% male, 48.5% female. From an age perspective, the group was dominated by the age group of 30 to 60. Such a group is consistent with the age profile of carers in Ireland where, according to the 2016 census (Census of Population 2016—Profile 9 Health, Disability and Carers ), 67% of carers were in this age group. All subjects had access to the internet, and all except one owned a mobile device. Perception of technologies was mixed: 76% of respondents claimed to trust technology; however, 86% expressed concerns regarding both security and privacy. Additionally, 65% of the subjects stated that they had the experience of caring for somebody, and 68% knew somebody being cared for; however, only 53% claimed they knew a carer.

##### Q2: Analysis of SUS Results

The results from questionnaire 2 were analysed and compared against several other questions for correlation, see Appendix C, for cross-correlation of relevant questions with SPSS scores.

##### Interface Preference, Line or Bar Graph

**Figure 16:**
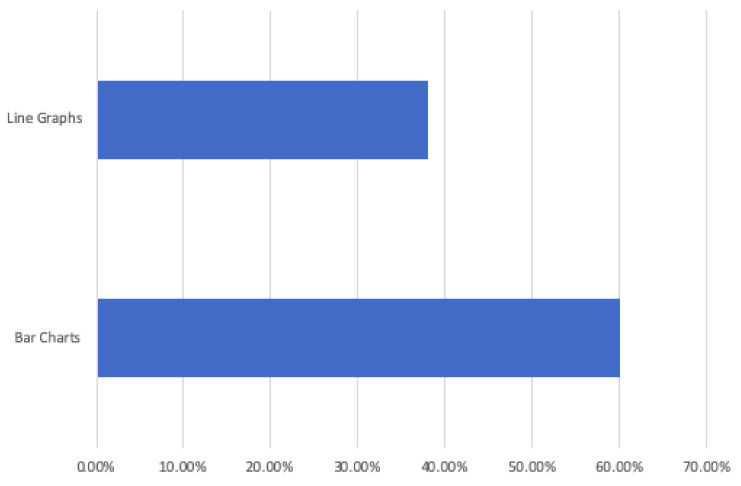
UI line or bar preference.

The analysis of this question’s results indicate that the 64 respondents preferred the bar graph, as 40 out of 64 or 60% chose this summarisation method over the alternative. The line graph UI had 26 out 64 respondents or 38% selecting this approach, see Figure 16. Respondents preferred the blue UI or bar graph format, which was reflected in an SUS score of 74%, whilst the line graph format or green UI obtained a lower SUS score of 71%. A score of 70% or greater is an acceptable score indicative that the interface is usable. Reasons attributed for not preferring the bar graph format included complexity (t(61) = 3.288; *p* < 0.01), a need for technical support (t(62) = 2.167; mboxemphp < 0.05), inconsistency (t(62) = 3.780; *p* < 0.05), cumbersome to use (t(62) = 3.780; *p* < 0.01) and a need for training (t(62) = 2.967; *p* < 0.01). For those who preferred the bar graph modality, an association between their experience as an active carer and their trust in technology was observed (*p* = 0.039). Likewise, there was also an association between their knowing a carer and their trust in technology (*p* = 0.026).

##### Trust of Technology

**Figure 17:**
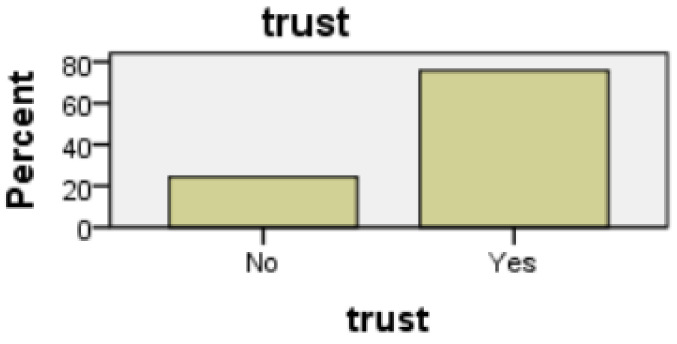
Qs 5a(pre) trust.

**Figure 18:**
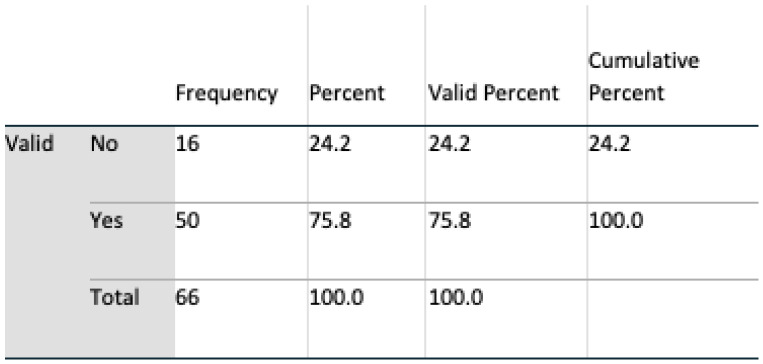
Qs 5a(pre) trust.

**Figure 19:**
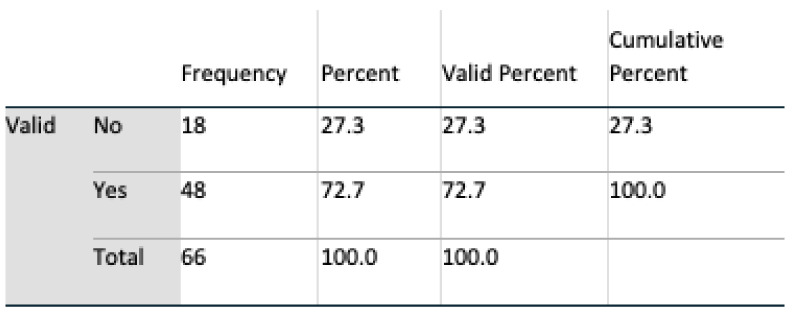
Qs 5a(pre) v Qs 16a(post) trust.

**Figure 20:**
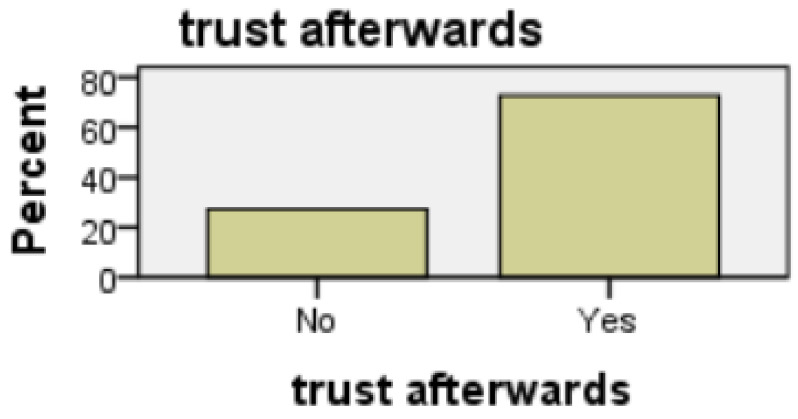
Qs 5a(pre) v Qs 16a(post) trust.

Those that expressed a trust in technology at the outset, were satisfied with both the line graph format (t(64) = 3.246, t < 0.01) and the bar graph format (t(63) = 2.278, *p* < 0.05). Furthermore, the line graph format was not perceived as inconsistent (t(64) = 2.141; *p* < 0.05) or cumbersome to use (t(64) = 2.129; *p* < 0.05). After evaluating both modalities of presentation, no statistical significance was observed in the case of the bar graph format, see Figure 17 and Figure 18. As before, those who did not trust technology considered the line graph format inconsistent (t(64) = 1.953, *p* < 0.05.) and cumbersome to use (t(64) = 1.983, *p* < 0.05). However, trust in technology decreased as a result of the evaluation of both presentation modalities; an association exists between those who, post evaluation, distrusted technology and would not allow a prospective carer to observe their day-to-day activities (*p* = 0.022, Fisher’s exact test). When participants answered question 5a, analysis suggests that there was a trust in technology, and that this decreased afterwards when the participants viewed the ADLs data visualised in both interfaces and answered question 16a, see Figure 19 and Figure 20.

##### Perception of Security

**Figure 21:**
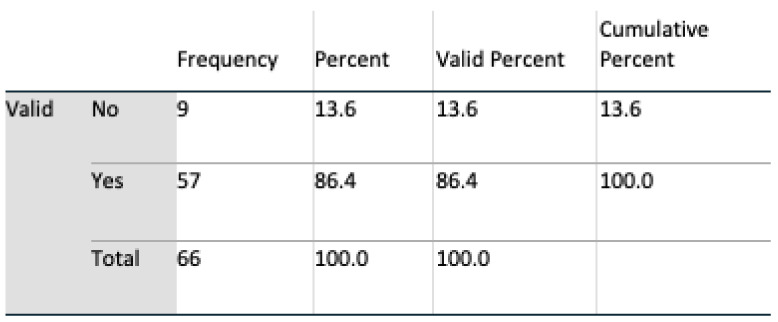
Qs 5b(pre) security.

**Figure 22:**
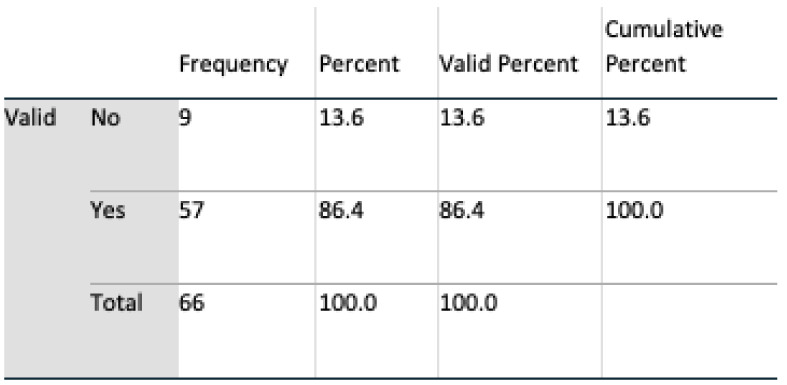
Qs 5b(pre) v Qs 16b(post) security.

Those who expressed no security concerns considered both the line graph format (t(64) = 2.122; *p* < 0.05) and the bar graph format (t(63) = 2.870; *p* < 0.01) as being sufficient. However, those who expressed concerns regarding security envisaged a need for learning prior to working with line graph, formats (t(64) = 1.672; *p* < 0.05). Attitudes to security remained broadly the same after completing the assessment with statistical significance being manifested as before. However, those with security concerns were more likely to know somebody being cared for (*p* = 0.024). No differences in attitudes to security in either of the pre- or post-evaluations were observed, see Figure 21 and Figure 22. Following analysis, there was no difference in security concerns after viewing the interfaces.

##### Perception of Privacy

**Figure 23:**
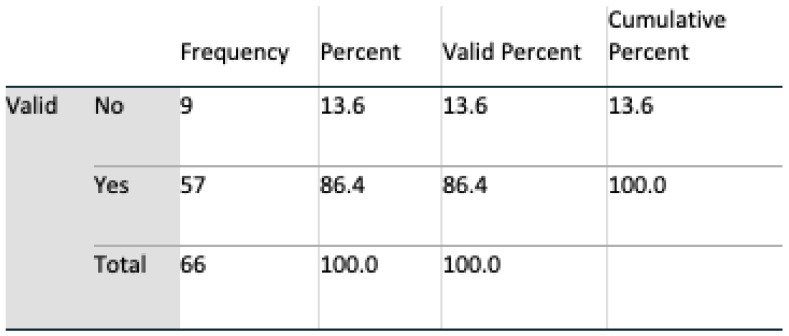
Q5c(pre) privacy.

**Figure 24:**
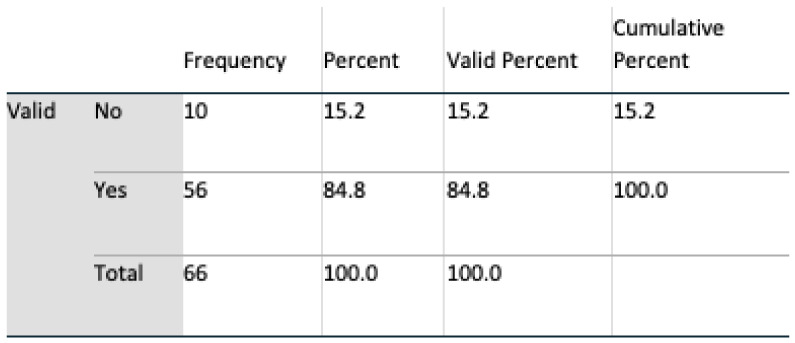
Qs 5c(pre) v Qs 16c(post) privacy.

Before undertaking the evaluation, those who expressed no concerns about privacy considered that the bar graph format did what they expected, t(63) = 2.384, *p* < 0.05. After completing the evaluation, attitudes to the bar graph format did not change (t(63) = 2.398; *p* < 0.05). However, in the case of the line graph format, attitudes did change, with subjects stating that this now did what they expected (t(64) = 2.166, *p* < 0.05). Knowing somebody who was being cared for influenced privacy perceptions (*p* = 0.047). However, privacy concerns marginally decreased following the completion of both evaluations, see Figure 23 and Figure 24. Fisher’s exact test indicated no significance.

##### The Carer Dimension

Those who knew a carer preferred the use of bar graphs, t(64) = 2.676, *p* < 0.01, and did not perceive the information presented as being inconsistent, t(64) = 1.711; *p* < 0.05. Learnability was not perceived as a problem in both bar (t(64) = 0.119, *p* < 0.05) and line graphs (t(64) = 0.068, *p* < 0.05). After assessing both modalities, security concerns increased for those who knew somebody being cared for (*p* = 0.024). Experience as a carer influenced perceptions of the line graph presentation modality. Those who had no experience as a carer considered this modality as being unnecessarily complex (t(62) = 1.917, *p* < 0.05) and inconsistent (t(62) = 2.537, *p* < 0.05).

##### Subjects as Potential Receivers of Care

When asked about their attitudes towards a potential caregiver monitoring their activities, those who expressed their willingness for allowing the monitoring of their activities considered both the line graph (t(63) = 2.421, *p* < 0.05 ) and bar graph (t(62) = 2.663, *p* < 0.01) presentation modality satisfactory, while expressing a preference for using the bar graph format (t(63) = 2.736, *p* < 0.01). As noted above, trust of technology was also manifested. For those who would not allow their activities be monitored, there was an association between knowing a carer and trust in technology (*p* = 0.048).

##### Gender Differences

Gender differences were not observed in all but one case. Males considered the presentation of information in the bar graph format inconsistent when compared to females—t(64) = 2.136, *p* < 0.05. There is no indication of why this was the case, and further investigation would be required in the future. Participant responses to questions 2 and 11 were compared, and when analysed it was concluded that male respondents thought the Loved-1 UI was more inconsistent than female respondents.

### 4.3. UI Research Contribution

These studies were conducted over a period of nine months, and the sample base constituency from which participants were drawn remained the same for both questionnaires. Previous usability studies recruited low participant numbers and based conclusions on limited response data analysis. The research studies presented in this paper recruited the largest participant sample base to date for caregiver UI usability analysis. This indicates a possible level of community willingness to participate in this type of research.

The aims and objectives from the first caregiver usability study were partially achieved following the analysis of 66 participant responses. The line and bar UIs were deemed by caregivers to be the easiest and most effective when remotely monitoring daily activity information. These results aligned with previously published UI studies. However, the second objective for this study required more investigation, and a review study could offer greater clarity regarding exactly which graph format combination caregivers preferred for usability and ease of use.

**Figure 25:**
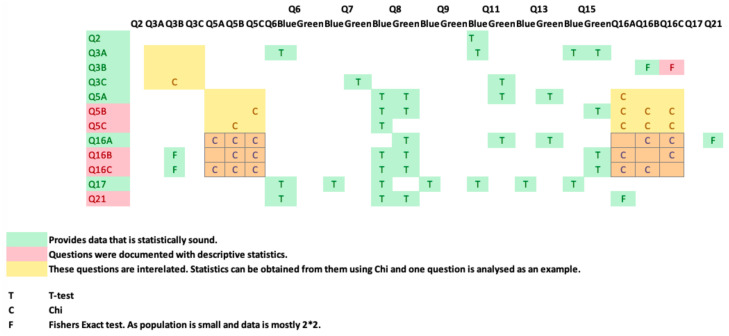
Questionnaire 2 SPSS Data Synthesis.

The second usability study analysed 64 participant responses regarding which UI combination, pie/line or pie/bar, was preferred by caregivers. Data analysis was conducted using SPSS v 24, *t*-tests, and chi-square tests, including Fisher’s exact test, see Figure 25. This study clarified interface usability, gathered demographic information on participant age profile, gender, experience of caring and confirmed whether the ADLs information being presented was understood by caregivers. The graph combination of pie/bar was chosen by caregivers as the easiest UI to understand when viewing different longitudinal timeline data; therefore, both study objectives were achieved.

These usability studies have contributed to UI research by reaffirming that line and bar graphs are the preferred choice for caregivers when viewing activity data. Furthermore, it was established that the graph combination of pie/bar is the preferred UI by caregivers for assimilating daily activity data when conducted over various timelines.

These results reaffirm previous usability conclusions and offer possible guidance for future researchers when developing interfaces for caregivers needs. Therefore, this paper and these UI usability results contribute significantly to the field of UI research for caregivers.

### 4.4. Respondent Feedback

The final question in Q1 requested feedback from the respondents and for them to rank the four UIs in order of preference, see results in Figure 15. Respondents ranked the interfaces but offered no feedback on their usability. Following analysis of the results, a follow-up questionnaire was conducted where respondents took the opportunity to offer feedback on the line (green) and bar (blue) Loved-1 UIs, and these comments were a valuable contribution towards any future iterations of this interface. The respondents comments could be separated into categories of general questionnaire layout, privacy issues and graph format choice.

General comments regarding the UIs included observations that an App would be appreciated, but that the tool itself was excellent, that it would be of great assistance to caregivers for the unobtrusive monitoring of an elder, easy to use on different mediums, and some wished it had been available to them when caring for a relative. Some respondents highlighted possible medical implications for some activities being presented, but the research objective was to explore the UI’s ability to convey ADLs information via the different graph formats and establish which the respondents found the easiest to understand.

When feedback on privacy was expressed, it comprised polar opposite opinions, either the monitoring of an elder with sensors within their own home was invading their personal privacy and removed the need for physical visits from a carer or relative, or respondents wanted a version of the Loved-1 system for their personal use in caring for a relative.

Regarding graph preferences, the comments indicate that the majority of respondents prefer the bar graph format. Some indicated a preference for histograms when viewing timeline data in comparison to line graphs, and others simply found the line graphs easier to understand.

## 5. Future Work

The information presented to caregivers must be easily understood and have the ability to provide longitudinal depictions of timelines, combined with daily activity summaries. The completion of certain ADLs may have a greater significance for the daily monitoring of some individuals, and interfaces should adapt to reflect evolving individual medical needs or situation. Future work is required to provide caregiver alerts considering type, frequency, priority and/or information content. Further investigations could incorporate refinements of the caregiver interfaces described in this paper to include respondent feedback. Such refinements would necessitate further usability studies, thereby ensuring their specific needs are met.

## 6. Conclusions

Ambient systems provide users with daily living assistance and present information in many formats. UIs affect system uptake, and this has had an impact upon usability; therefore, the summarisation of data for caregivers requires careful consideration. This paper conducted two research studies into interfaces for the presentation of ADLs to caregivers and recruited a significantly higher number of participants than had been involved in any similarly cited UI research. The first study as documented in this paper presented four UIs to caregivers for the elderly in order to establish which one was the easiest to use, provides them with information relevant to their needs in a manner that is timely, responsive to events and offered caregivers an unobtrusive view into the daily lives of their loved ones. The results from this questionnaire verified that respondents preferred a combination interface, but this was not affirmed by the SUS score. Research studies indicate that line, bar and pie charts are the most easily understood formats for displaying ADLs to users. A combination approach optimises the features from each format to maximise comprehension.

Subsequently, a second review study was undertaken in order to clarify which combination of graphs was preferred by caregivers and which format, bar or line graphs, was the easiest to understand. The research hypotheses were proven when respondents were specifically asked to choose between line and bar graphs regarding which format combination they preferred. This research study can, therefore, confirm that a majority of caregivers preferred the bar graph format when compared to those who chose the line graph format. This result complies with previous research studies. Additionally verified in this study, caregivers preferred a pie graph format for short time interval visualisations in combination with a bar graph format, which was the most easily understood method for representing extended timeline intervals. Again, this is in accordance with previous research findings.

## Abbreviations

The following abbreviations are used in this manuscript:

AAL	Ambient Assisted Living
ADLs	Activities of Daily Living
AL	Assisted Living
AmI	Ambient Intelligence
HCC	Human Centered Computing
HCI	Human Computer Interaction
ICT	Information and Communications Technology
LD	Linear Dichroism
SUS	System Usability Scale
UCD	University College Dublin
UI	User Interface
URL	Uniform Resource Locator
UX	User eXperience

## Appendix C. Q2 Cross-Correlation of Questions—Evaluation of User Interface (UI) Usability for Caregivers Monitoring an Elder’s Activities of Daily Living (ADLs) UCD Ethics Reference no LS-19-67

**Interface Preference, Line (Green) or Bar (Blue) Graph**

**Qs 17: Please choose the UI you preferred?, Line (Green) or Bar (Blue) Graph**

**Versus**

*Qs 6: I think that I would like to use this UI frequently?* (Blue)

T(62) = 2.740; *p* < 0.01

Those who selected Blue would like to use this UI frequently.

*Qs 7: I found the UI unnecessarily complex?* (Blue)

T(61) = 3.288; *p* < 0.01

Those who selected the Green UI were more like to find the Blue interface more complex.

*Qs 8: The UI does everything I would expect it to do?* (BLUE)

T(61) = 1.755; *p* <0.05

Those who selected Blue consider the UI to do everything they would expect to.

*Qs 9: I think that I would need the support of a technical person to use this interface?* (BLUE)

T(62) = 2.167; *p* < 0.05

Those who selected Green were more inclined to need technical support for the Blue UI.

*Qs 11: I thought there was too much inconsistency in this UI?* (BLUE)

T(62) = 3.780; *p* < 0.05

Those who selected Green thought the Blue UI site inconsistent.

*Qs 13: I found the UI very cumbersome to use?* (Blue)

T(62) = 3.780; *p* < 0.001

Those who selected Green thought the Blue UI very cumbersome.

*Qs 15: I needed to learn a lot of things before I could get going with this UI?* (Blue)

T(62) = 2.967; *p* < 0.01.

Those who selected Green felt they needed to learn a lot before they could use the Blue interface.

**Trust of Technology**

**Qs 5A: Do you trust technology?**

**Versus**

*Qs 8: The UI does everything I would expect it to do?* (Blue)

T(63) = 2.278; *p* < 0.05.

Those who trusted technology thought that the Blue UI does everything I would expect it to do.

*Qs 8: The UI does everything I would expect it to do?* (Green)

t(64) = 3.246; t < 0.01.

Those who trusted technology thought that the Green UI does everything I would expect it to do.

*Qs 11: I thought there was too much inconsistency in this UI?* (Green)

T(64) = 2.141; *p* < 0.05.

Those who trusted technology did NOT think there was too much inconsistency in this UI

*Qs 13: I found the UI very cumbersome to use?* (Green)

T(64) = 2.129; *p* < 0.05

Those who trusted technology did NOT find the UI very cumbersome to use.

*Qs 21: If you were being cared for, would you be happy for your carer to know your activities as specified in the previous screens?*

X2 = 4.104, df = 1, *p* < 0.05

If technology was trusted, participants were more likely to allow the carer to know activities. Fishers Exact test, reports no significance

When participants answered question 5a, analysis suggests that there was a trust in technology, and that this decreased afterwards when the participants viewed the ADLs data visualised in both interfaces and answered question 16a, see Figure 19 and Figure 20.

**Qs 16a: Do you trust technology?** AFTERWARDS

**Versus**

*Qs 8: The UI does everything I would expect it to do?* (Green)

T(64) = 2.473; *p* < 0.05

Those who trust technology consider that the Green UI does everything I would expect it to do.

*Qs 11: I thought there was too much inconsistency in this UI?* (Green)

T(64) = 1.953; *p* < 0.05.

Those who do NOT trust technology thought there was too much inconsistency in this UI.

*Qs 13: I found the UI very cumbersome to use?* (Green)

T(64) = 1.983; *p* < 0.05.

Those who NOT trust technology found the Green UI MORE cumbersome to use.

*Qs 21: If you were being cared for, would you be happy for your carer to know your activities as specified in the previous screens?*

Fishers Exact Test – *p* = 0.022

T(63) = 2.043; *p* < 0.05.

Those who trust technology would be happy for their carer to know their activities

**Perception of Security**

**Qs 5B: Do you have any security concerns regarding technology?**

**Versus**

*Qs 8: The UI does everything I would expect it to do?* (Blue)

T(63) = 2.870; *p* < 0.01

Those who has NO security concerns thought that the Blue UI does everything I would expect it to do

*Qs 8: The UI does everything I would expect it to do?* (Green)

T(64) = 2.122; *p* < 0.05

Those who had NO security concerns thought that the Green UI does everything I would expect it to do

*Qs 15: I needed to learn a lot of things before I could get going with this UI?* (Green)

T(64) = 1.672; *p* < 0.05.

Those who had security concerns thought that they needed to learn a lot of things before I could get going with this Green UI

*Qs 16B: Do you have any security concerns regarding technology* (AFTERWARDS)?

X2 = 5.834, df = 1, *p* < 0.5 or X2 (1, N = 66) = 5.834 *p* < 0.05.

Fishers Exact test: *p* = 0.024

Conclude that the association is significant.

T(64) = 2.032; *p* < 0.05.

Those who knew somebody who is being cared for had security concerns (AFTERWARDS).

Following analysis there was no difference in security concerns after viewing the interfaces.

**Qs 16B: Do you have any security concerns regarding technology**

**Versus**

*Qs 3B: Know someone who is being cared for*

Fisher says *p* = 0.024

Those who have security concerns are more likely to know somebody being cared for.

*Qs 8: The UI does everything I would expect it to do?* (Blue)

T(63) = 2.384; *p* < 0.05.

Those who have NO security concerns tended to view the Blue UI as doing everything they expected.

*Qs 8: The UI does everything I would expect it to do?* (Green)

T(64) = 2.122; *p* < 0.05.

Those who have NO security concerns tended to view the (Green) UI as doing everything they expected

*Qs 15: I needed to learn a lot of things before I could get going with this UI?* (Green)

T(64) = 2.143; *p* < 0.05.

Those who had security concerns felt they needed to learn a lot of things.

**Perception of Privacy**

**Qs 3b: Do you Know someone who is being cared for?**

**Versus**

*Qs 16C: Do you have any privacy concerns regarding technology* (AFTERWARDS)?

T(64) = 1.793; *p* < 0.05.

X2 = 4.315, df = 1, *p* < 0.05 Fishers Exact test indicated no significance.

Those who knew somebody who is being cared for had privacy concerns (AFTERWARDS).

**Qs 5c: Do you have any privacy concerns regarding technology?**

**Versus**

*Qs 8: The UI does everything I would expect it to do?*(Blue)

T(63) = 2.384; *p* < 0.05.

Those who did not have privacy concerns thought the Blue UI did everything they expected.

**Qs 16c: Do you have any privacy concerns regarding technology**

**Versus**

The same questions were used for correlating 3b.

*Qs 3b: Know someone who is being cared for?*

T(64) = 2.116; *p* < 0.05.

X2 = 4.315. df = 1, *p* < 0.05

Fishers test says: *p* = 0.047.

Those who have privacy concerns were more likely to have someone being cared for.

*Qs 8: The UI does everything I would expect it to do?*(Blue)

T(63) = 2.398; *p* < 0.05.

Those who have NOT any privacy concerns believed the Blue UI does everything I would expect

*Qs 8: The UI does everything I would expect it to do?* (Green)

T(64) = 2.166; *p* < 0.05

Those who have NOT any privacy concerns believed the Green UI does everything I would expect

**The Carer Dimension**

**Qs 3a: Experience as a carer? Know someone who is a carer – Yes/No Versus**

This question was compared with several other questions for comparison.

*Qs 6: I think that I would like to use this UI frequently?* (Blue)

T(64) = 2.676; *p* < 0.01.

Those who knew a carer preferred to use this UI Blue frequently.

*Qs 11: I thought there was too much inconsistency in this UI?* (Blue)

T(64) = 1.711; *p* < 0.05.

Those who knew a carer did NOT think this Blue UI was too inconsistent.

*Qs 15: I needed to learn a lot of things before I could get going with this UI?* (Blue)

T(64) = 0.119; *p* < 0.05

Those who knew a carer did NOT think they needed to learn a lot of things before getting going with the Blue UI.

*Qs 15: I needed to learn a lot of things before I could get going with this UI?* (Green)

T(64) = 0.068; *p* < 0.05.

Those who knew a carer did NOT think they needed to learn a lot of things before getting going with the Green UI.

**Experience as a carer**

All the Q3 questions are interrelated.

**Qs 3a: Know someone who is a carer?**

**Versus**

*Qs 3b: Do you Know someone who is being cared for?*

Fisher Exact test: *p* = 0.002

If the respondent was a carer, they are more likely to know somebody being cared for.

*Qs 3c: Is or have been an active carer?*

*p* = 0.001

If the respondent knows a carer, they are more likely to know somebody being cared for.

**Know someone who is being cared for?**

**Qs 3B: Know someone who is being cared for?**

**Versus**

*Qs 3a: Know someone who is a carer?*

Fisher Exact test: *p* = 0.002

If respondents know somebody being cared for, they will also know a carer.

**Qs 3c: Is or have been an active carer**

**Versus**

*Qs 3a: Know someone who is a carer?*

Fisher = 0.001.

**Qs 3c: Is or have been an active carer**

**Versus**

*Qs 7: I found the UI unnecessarily complex?* (Green)

T(62) = 1.917, *p* < 0.05

Those who were NOT active carers thought the Green UI unnecessarily complex.

*Qs 11: I thought there was too much inconsistency in this UI?* (Green)

T(62) = 2.537, *p* < 0.05

Those who were NOT active carers thought the Green UI too inconsistent.

**Subjects as potential receivers of care**

**Qs 21: If you were being cared for, would you be happy for your carer to know your activities as specified in the previous screens?**

**Versus**

*Qs 6: I think that I would like to use this UI frequently?*(Blue)

T(63) = 2.736; *p* < 0.01

Those who were happy about carer would use the Blue UI frequently

*Qs 8: The UI does everything I would expect it to do?*(Blue)

T(62) = 2.663; *p* = 0.01

Those who were happy agreed that the Blue UI did everything expected.

*Qs 8: The UI does everything I would expect it to do?*(Green)

T(63) = 2.421; *p* < 0.05

Those who were happy agreed that the Green UI did everything expected.

*Qs 16a: Do you trust technology*? AFTERWARDS

T(63) = 2.568; *p* < 0.05

Fishers Exact test: *p* = 0.022.

Those who were happy did trust technology

**Gender Differences**

**Qs 2: What is your Gender? Versus**

*Qs11: I thought there was too much inconsistency in this UI?* T(64) = 2.136; *p* < 0.05.
